# TcbHLH14 a Jasmonate Associated MYC2-like Transcription Factor Positively Regulates Pyrethrin Biosynthesis in *Tanacetum cinerariifolium*

**DOI:** 10.3390/ijms24087379

**Published:** 2023-04-17

**Authors:** Tuo Zeng, Qin Yu, Junzhong Shang, Zhizhuo Xu, Li Zhou, Wei Li, Jinjin Li, Hao Hu, Liyong Zhu, Jiawen Li, Caiyun Wang

**Affiliations:** 1 School of Life Sciences, Guizhou Normal University, Guiyang 550025, China; 2 National Key Laboratory for Germplasm Innovation & Utilization of Horticultural Crops, College of Horticulture & Forestry Sciences, Huazhong Agricultural University, Wuhan 430070, China

**Keywords:** function analysis, MYC2-like transcription factors, pyrethrins, *Tanacetum cinerariifolium*

## Abstract

Natural pyrethrins have high application value, and are widely used as a green pesticide in crop pest prevention and control. Pyrethrins are mainly extracted from the flower heads of *Tanacetum cinerariifolium*; however, the natural content is low. Therefore, it is essential to understand the regulatory mechanisms underlying the synthesis of pyrethrins through identification of key transcription factors. We identified a gene encoding a MYC2-like transcription factor named *TcbHLH14* from *T. cinerariifolium* transcriptome, which is induced by methyl jasmonate. In the present study, we evaluated the regulatory effects and mechanisms of TcbHLH14 using expression analysis, a yeast one-hybrid assay, electrophoretic mobility shift assay, and overexpression/virus-induced gene silencing experiments. We found that TcbHLH14 can directly bind to the cis-elements of the pyrethrins synthesis genes *TcAOC* and *TcGLIP* to activate their expression. The transient overexpression of TcbHLH14 enhanced expression of the *TcAOC* and *TcGLIP* genes. Conversely, transient silencing of *TcbHLH14* downregulated the expression of *TcAOC* and *TcGLIP* and reduced the content of pyrethrins. In summary, these results indicate that the potential application of TcbHLH14 in improving the germplasm resources and provide a new insight into the regulatory network of pyrethrins biosynthesis of *T. cinerariifolium* to further inform the development of engineering strategies for increasing pyrethrins contents.

## 1. Introduction

*Tanacetum cinerariifolium* is a perennial herb of the genus *Tanacetum* belonging to the Asteraceae family, which is an economically important species and widely used for the extraction of essential oils, mainly comprising pyrethrins that have fast-acting, broad-spectrum insecticidal activities with high efficiency. Most importantly, pyrethrins have a short half-life (approximately 2 h) and thus do not leave residue or enrich the environment, offering a safe alternative to many commonly used synthetic pesticides that have potential to cause harm to human health and the ecological environment [1,2]. Thus, pyrethrins have emerged as the most promising botanical insecticides. However, the naturally available amount of pyrethrins in the flower heads is too low with the content of 0.36% to 1.35% in *T. cinerariifolium* populations [3], thereby limiting the planting and application. Therefore, it is necessary to study the biosynthesis and regulation of pyrethrins to improve the productivity.

Natural pyrethrins are composed of six monoterpene esters (Figure S1), which are synthesized from two monoterpenoid acids (chrysanthemic acid and pyrethric acid) and three rethrolone-type oxylipin alcohols (cinerolone, jasmolone, and pyrethrolone) [4]. The two monoterpenoid acid precursors are irregular monoterpenes containing a cyclopropane ring structure synthesized via the 2-C-methyl-d-erythritol 4-phosphate pathway [5]. Two molecules of dimethylallyl pyrophosphate are successively synthesized to chrysanthemyl diphosphate (CDP) and chrysanthemol under the continuous catalysis of chrysanthemol synthase (CHS) [6]. Subsequently, CDP and chrysanthemol are oxidized by alcohol dehydrogenase and aldehyde dehydrogenase (ALDH) to produce *trans*-chrysanthemic acid [7], and are further catalyzed to pyrethric acid by chrysanthemol 10-hydroxylase and 10-carboxychrysanthemic acid 10-methyltransferase [8]. The alcohol moiety substrate rethrolones are at least partially obtained from the jasmonate (JA) biosynthesis pathway [4,9] in the conversion of linolenic acid to *oxo*-phytodienoic acid, including lipoxygenase, allene oxide synthase, and allene oxide cyclase (AOC) as the key molecules involved in JA synthesis [10,11]. Jasmone hydroxylase converts jasmone to jasmolone [12], and then pyrethrolone synthase catalyzes jasmolone to pyrethrolone [13]. Finally, GDSL lipase-like protein (GLIP) esterifies the acid and alcohol moieties into pyrethrins [14].

The bHLH (basic Helix–Loop–Helix) transcription factor family represents one of the most extensive families of transcription factors in plants. This family comprises two distinct regions with different functions: the basic region and the HLH region. The basic region, which specifically binds to the E-box (CANNTG) or G-box (CACGTG) of the DNA sequence [15]. Conversely, the HLH region has the capability to form heterodimers or homodimers with other proteins [16]. bHLH transcription factors play their roles mainly through the JA signal pathway in plants, and inhibit/activate the expression of JA-responsive genes [17,18], in turn regulating plant growth and development, the stress response, and synthesis of secondary metabolites. CrMYC2 can upregulate the expression of octadecanoid-response Catharanthus AP2 proteins (ORCA) to increase the biosynthesis of terpenoid indole alkaloids (TIAs) in *Catharanthus roseus* [19]. The heterologous expression of AtMYC2 can enhance the transcription of genes involved in the MEP pathway, thereby accumulating more Abietane diterpenes in *Salvia striata* [20]. AabHLH112 promotes artemisinin biosynthesis in *Artemisia annua* [21], and AabHLH1 responds to ABA and activates the expression of artemisinin synthesis-related genes, thereby increasing artemisinin production [22].

We screened TcbHLH14 from *T. cinerariifolium* as a TF acting in response to the methyl jasmonate (MeJA) signal, and previous studies also found that MeJA can up-regulate the expression of genes related to pyrethrins synthesis to ultimately increase the pyrethrins content [12]. Together, these findings suggest that TcbHLH14 may be involved in the regulation of pyrethrins biosynthesis mediated by MeJA. To further elucidate the mechanism of pyrethrins transcriptional regulation, in the present study, we explored the regulatory effect of the TF TcbHLH14 induced by MeJA on the key genes of pyrethrins synthesis. These results have important theoretical and practical significance for developing an appropriate genetic engineering strategy to enhance the biosynthesis of pyrethrins and cultivate varieties with high pyrethrins contents.

## 2. Results

### 2.1. Domain and Homology Analysis of the TcbHLH14 Gene

**Figure 1:**
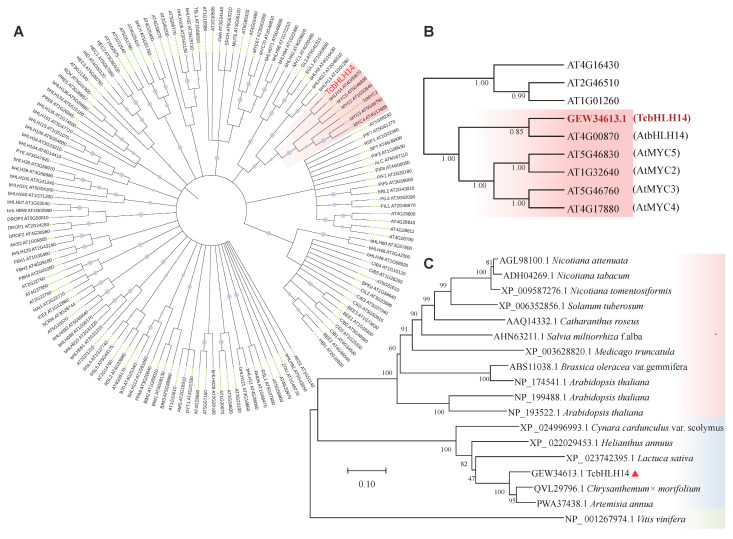
Phylogenetic analysis of TcbHLH14 and other bHLH proteins. (**A**) Phylogenetic analysis of bHLH transcription factors of *A. thaliana* and *T. cinerariifolium*. (**B**) The branch including TcbHLH14 and AtbHLH14 is marked with a pink background; TcbHLH14 is displayed in red font. (**C**) Phylogenetic analysis of TcbHLH14 and bHLH proteins of other plants; TcbHLH14 is marked with the red triangle; the green box indicates Vitaceae, the blue box represents Asteraceae, and the pink box includes Brassicaceae Burnett, Fabaceae, Apocynaceae, and Solanaceae. The tree was constructed by the neighbor-joining method with 1000 bootstrap replicates.

The bHLH amino acid sequence of *A. thaliana* was downloaded from the NCBI database, and the phylogenetic tree of *TcbHLH14* pyrethrum and Arabidopsis bHLH TFs was constructed (Figure 1A). The results showed that Arabidopsis AtbHLH14 has the closest genetic relationship with pyrethrum bHLH (GEW34613.1) (Figure 1B), indicating that *T. cinerariifolium* GEW34613.1 may have a function similar to AtbHLH14. Therefore, the ORF sequence of GEW34613.1 was cloned and named *TcbHLH14*.

To further explore the evolutionary relationship of the *T. cinerariifolium* TF TcbHLH14, a molecular evolutionary tree was established based on the amino acid sequences of TcbHLH14 and bHLH_MYC proteins of other plant species using MEGAX software. Among the 18 selected plants, bHLH_MYC proteins mainly clustered into three independent evolutionary branches (Figure 1C): grape, Asteraceae, and other (Cruciferae, Leguminosae, Labiatae, Apocynaceae, Solanaceae) branches, marked with green, blue, and pink background frames, respectively. *T. cinerariifolium*, *Chrysanthemum × morifolium*, and *A. annua* in the Asteraceae branch clustered in the same subfamily, which indicated that they had high homology, revealing that TcbHLH14 protein and its homologs are closely related within the Asteraceae family.

The bHLH proteins of other species with high similarity to the pyrethrum TF TcbHLH14 were searched in the NCBI/Blast database (https://www.ncbi.nlm.nih.gov/Blast.cgi (accessed on 26 September 2022)). The selected homologous proteins were compared with TcbHLH14 proteins using MUSCLE and BoxShade software. The TcbHLH14 protein was compared with *A. annua*, PWA37438.1, and artichoke (*Cynara cardunculus* var). The bHLH proteins in Scolymus (*Cynara cardunculus*, XP_024996993.1), chrysanthemum (*Chrysanthemum* × *morifolium*, QVL29796.1), sunflower (*Helianthus annuus*, XP_022029453.1), and lettuce (*Lactuca sativa*, XP_023742395.1) were highly similar, reaching 88.54%, 65.12%, 88.78%, 71.43%, and 65.80% similarity, respectively. Compared to TcMYC2 and AtMYC2, TcbHLH14 displays a significant deletion in the bHLH MYC-N domain. Additionally, the N-terminal region of TcbHLH14 exhibits numerous deleted protein residues in contrast to AtbHLH14 (Figure S2).

### 2.2. TcbHLH14 Gene Expression in Tissues

**Figure 2:**
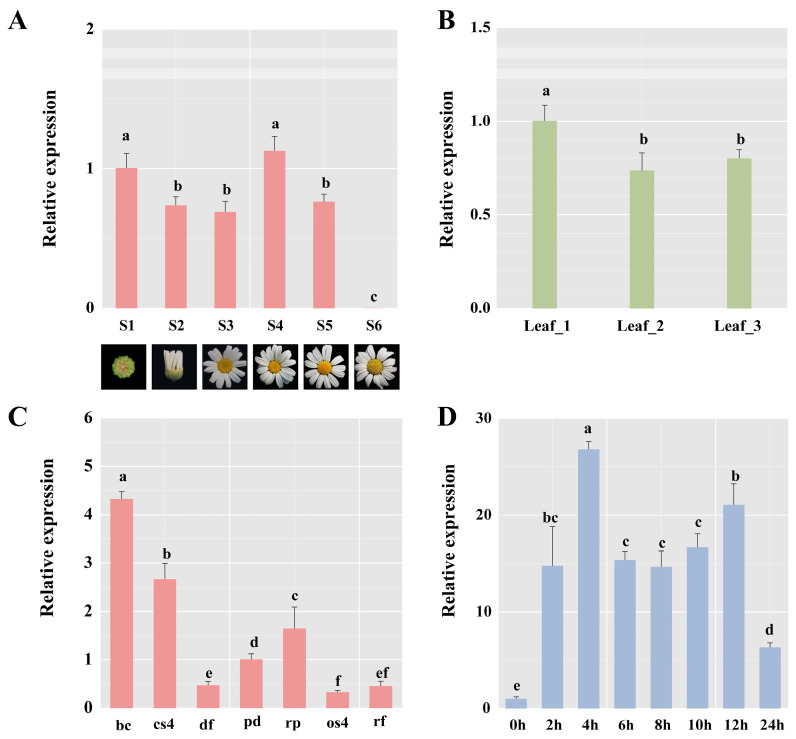
Expression distribution of *TcbHLH14* in *T. cinerariifolium* tissues. RT-qPCR analysis of *TcbHLH14* transcript abundance in leaf and flower tissues at different developmental stages. (**A**) Stage 1 (S1, bud), Stage 2 (S2, half open white petalled ray florets), Stage 3 (1st row of the yellow disc-florets), Stage 4 (S4, half rows of the yellow disc-florets), Stage 5 (S5, all rows of the yellow disc-florets open), Stage 6 (S6, withering flowers). (**B**) Leaf 1 (L1, width of 5–10 mm), Leaf 2 (L2, width of 15–20 mm), Leaf 3 (L3, width > 25 mm). (**C**) Expression level normalized against the pedicel transcript level in the S2 flower stages (bc: bract; pd: pedicel; rp: receptacle; rf: ray floret; df: disc floret) and S4 flower stages (os4: fully open ray floret; cs4: unopened ray floret). (**D**) Expression analysis of *TcbHLH14* after MeJA treatment of *T. cinerariifolium* leaves. Different lower-case letters indicate significant differences (*p* < 0.05) from one-way ANOVA followed by Tukey post hoc test.

The transcriptional level of *TcbHLH14* in the flowers, leaves, and floral organs of *T. cinerariifolium* at different developmental stages was detected by RT-qPCR. The expression level of *TcbHLH14* in *T. cinerariifolium* decreased during the S1–S3 stages of flower development, reached the peak in the S4 stage, decreased gradually due to the gradual withering of flowers, and was barely expressed by the S6 stage (Figure 2A). In addition, the expression level of *TcbHLH14* was the highest in the L1 stage, followed by the L3 stage, and was the lowest in the L2 stage (Figure 2B). After dissecting the flower head of the S2 stage into five parts (ray floret, disc floret, bract, receptacle, and pedicel) and the S4 stage into two parts (ray floret and disc floret), we found that the expression level of *TcbHLH14* was the highest in the S2 bract, followed by the S4 closed-tube flower (Figure 2C).

MeJA is an indispensable signaling molecule in plants, which not only regulates secondary plant metabolism but also participates in plant defense responses to biotic and abiotic stresses. We found that the *TcbHLH14* gene could be induced by MeJA at different time periods (Figure 2D).

### 2.3. Subcellular Localization of TcbHLH14

**Figure 3:**
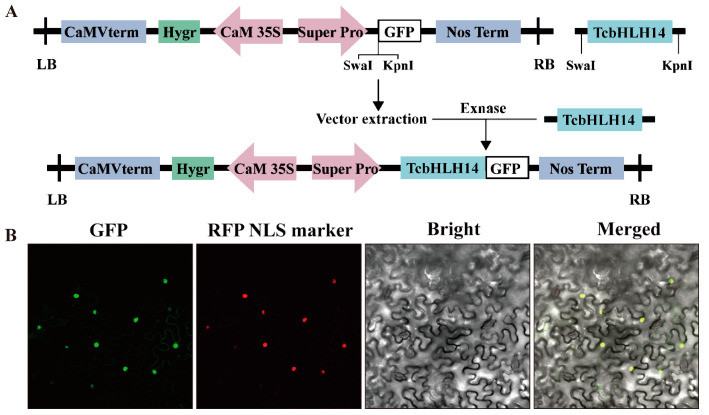
Subcellular localization of TcbHLH14. (**A**) Schematic diagram of vector construction. TcbHLH14 was inserted into the pSuper1300-GFP vector driven by the Super promoter. (**B**) Green fluorescence emitted by the TcbHLH14::green fluorescent protein (GFP) fusion protein driven by the Super promoter. RFP NLS: the nuclear-labeled fusion protein emits red fluorescence; Bright: ordinary light source; Merged: fusion of green and red fluorescence and ordinary light.

To identify the subcellular localization of TcbHLH14 protein, the TcbHLH14-GFP fusion protein, driven by the Super promoter (Figure 3A), was transiently expressed in *N. benthamiana* and detected by laser confocal scanning microscopy. The green fluorescence signal detected in the TcbHLH14-GFP fusion protein overlapped with the red fluorescence signal in the nuclear localization plasmid under specific excitation light, indicating that TcbHLH14 is a nuclear-localized protein, which corresponds with exerting its function as a TF in regulating gene expression (Figure 3B).

### 2.4. TcbHLH14 Directly Binds to the Promoters of the TcAOC and TcGLIP Genes

**Figure 4:**
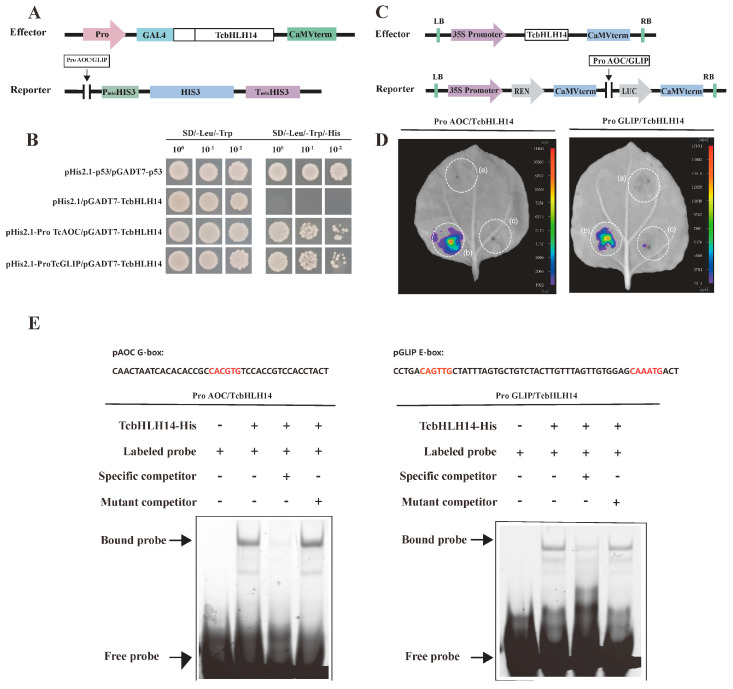
TcbHLH14 directly binds to the promoters of *TcAOC* and *TcGLIP*. (**A**) Diagram of Y1H vectors, Effector: TcbHLH14 was inserted into the pGADT7 vector, Reporters: the promoter of TcAOC/TcGLIP was inserted into the pHis2.1 vector (**B**) Y1H assay showing TcbHLH14 binding to TcAOC/TcGLIP promoter fragments. Ten-fold dilution series of yeast solution cultured on SD/-Leu/-Trp and SD/-Leu/-Trp/-His culture plates at 30 °C for 4 days. pHis2.1/pGADT7-TcbHLH14: negative control; pHis2.1-p53/pGADT7-p53: positive control. (**C**) Diagram of LUC vectors, Effector: TcbHLH14 was inserted into the pGreen-62-SK vector driven by the CAMV 35S promoter; Reporters: the promoters of TcAOC/TcGLIP were inserted into the pGreen II-0800-LUC vector and drove LUC RNA translation. (**D**) Transient expression assays showing TcbHLH14 binding to the TcAOC/TcGLIP promoters and promotion of Luc expression a: pGreen II 62-SK; b: pGreen II 62-SK::TcbHLH14/pGreen II 0800-LUC::promoter TcAOC/TcGLIP; c: pGreen II 62-SK/pGreen II 0800-LUC::promoter TcAOC/TcGLIP. (**E**) EMSA of interaction between TcbHLH14 and the promoters of TcAOC/TcGLIP. The probe was designed from a fragment of the promoter containing E-box or G-box motifs. Purified His-tagged TcbHLH14 protein solution was incubated with 10 µM FAM-labeled probe. Non-labeled specific cold probes and mutated cold probes at 50-fold dilution were used for the competition test. The shifted bands and free probe are indicated with an arrow.

The yeast one-hybrid (Y1H) assay, luciferase reporter assay, and EMSA were used to verify whether TcbHLH14 interacts with the promoters of the *TcAOC* and *TcGLIP* genes in the pyrethrins biosynthesis pathway. The promoter of each gene was cloned and inserted into the pHis2.1 vector to generate reporter vectors. *TcbHLH14* was fused to the GAL4 activation domain to generate the effector construct pGADT7::*TcbHLH14* (Figure 4A). After co-transformation of pGADT7::*TcbHLH14*/pHis2.1::pro*TcAOC*/pro*TcGLIP*, the Y187 yeast strain showed normal growth on the three-deficiency culture plate SD/-Trp/-Leu/-His, indicating that there was an interaction between TcbHLH14 and the *TcAOC* and *TcGLIP* promoters (Figure 4B). To verify the interaction of TcbHLH14 with the *TcAOC* and *TcGLIP* promoters in vivo, reporter vector (*TcAOC*/*TcGLIP*pro:LUC) and effector vector (35S: *TcbHLH14*) were transiently coexpressed in *N. benthamiana* leaves (Figure 4C). The firefly luciferase activity was significantly higher than that of the control group after co-infection of pGreenII62-SK::TcbHLH14/pGreenII0800-LUC::pTcAOC/TcGLIP into *N. benthamiana* leaves, indicating that the TF TcbHLH14 could activate the transcription of *TcAOC* and *TcGLIP* gene promoters (Figure 4D). In the EMSA, TcbHLH14-His was transferred into the *E. coli* Rosetta strain for induction and purification. A labeled probe containing the G/E-box motif was designed in the *TcAOC/TcGLIP* promoter regions and the EMSA results showed that TcbHLH14 protein could bind to the probe along with a significant blocking band. When a 50× competitive cold probe was added, the blocking binding band was close to zero, whereas when the 50× mutant cold probe was added, the blocking band was restored, indicating that TcbHLH14 could bind to the G/E-box element in the *TcAOC/TcGLIP* promoters (Figure 4E). In summary, these assays collectively demonstrated that TcbHLH14 can directly bind to the *TcAOC* and *TcGLIP* promoters to activate these pyrethrin biosynthesis genes.

### 2.5. Transient Overexpression and VIGS of TcbHLH14 in T. cinerariifolium

**Figure 5:**
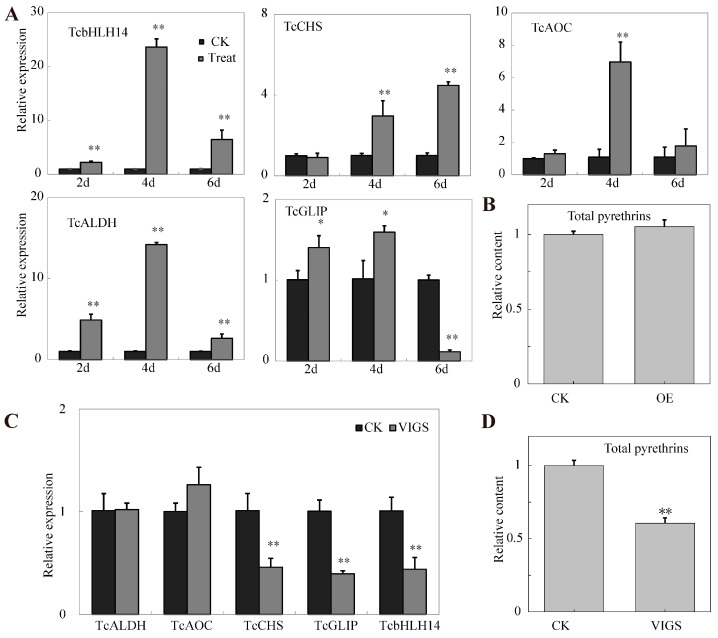
Determination of pyrethrin biosynthesis-related gene expression and pyrethrin content in the leaves with transient overexpression and silencing of *TcbHLH14*. (**A**) Expression levels of *TcGLIP*, *TcALDH*, *TcAOC*, *TcCHS*, and *TcbHLH14* genes in pyrethrum leaves after transient overexpression of *TcbHLH14* for 2, 4, and 6 days. (**B**) After transient overexpression of *TcbHLH14* for 4 days, the content of total pyrethrins in the leaves was measured. (**C**) Expression levels of *TcGLIP*, *TcALDH*, *TcAOC*, *TcCHS*, and *TcbHLH14* genes in the leaves after transient VIGS silencing of *TcbHLH14*. (**D**) Content of the total pyrethrins in the leaves after transient silencing of *TcbHLH14*. Data are presented as means ± SD of three technical replicates. Statistical significance was assessed with Student’s *t*-test (** *p* < 0.01; * *p* < 0.05).

To further study the effect of TcbHLH14 on pyrethrins biosynthesis, pGreenII62SK::TcbHLH14 was transiently transformed into *T. cinerariifolium* leaves for overexpression. The expression levels of pyrethrins biosynthesis pathway genes *TcGLIP, TcAOC*, *TcCHS*, *TcALDH1,* and the target gene *TcbHLH14* were detected by RT-qPCR. Compared with that of the control, the expression of *TcbHLH14* was significantly up-regulated on the second, fourth, and sixth day after transfection. As a whole, the expression profile showed a changing trend of rising first and then falling. The expression levels of *TcGLIP*, *TcAOC*, *TcCHS*, and *TcALDH1* were significantly increased on the fourth day, and the expression of *TcAOC*, *TcCHS*, and *TcALDH1* was up-regulated by more than 2.96 times. The *TcGLIP* expression level increased by 1.57 times (Figure 5A). However, no significant difference (*p* > 0.05) was observed in the total pyrethrins content between the control and treatment groups (Figure 5B, Table S1).

To further verify the role of *TcbHLH14* in pyrethrins biosynthesis, pTRV2::TcbHLH14 and pTRV1 virus-mediated silencing vectors were transiently transformed into pyrethrum leaves, and the expression levels of the above five genes were determined. The results of RT-qPCR showed that the expression level of *TcbHLH14* in the VIGS group was significantly lower than that of the control. The expression of *TcGLIP* and *TcCHS* was down-regulated by more than 2 times, while the expression of *TcAOC* and *TcALDH1* was slightly up-regulated after *TcbHLH14* silencing, but there was no significant change (Figure 5C). Moreover, the content of pyrethrins in the leaves decreased after *TcbHLH14* silencing (Figure 5D, Table S2).

## 3. Discussion

The plant hormone MeJA is a ubiquitous and conservative plant secondary metabolic elicitor [23]. Previous studies have found that MeJA can effectively promote pyrethrins accumulation [24], but its regulatory mechanism is unclear. To explore the effect of JA on pyrethrins synthesis, transcriptome analysis of pyrethrum leaves treated with MeJA was performed, which showed that MeJA could trigger transcriptional re-editing, resulting in extensive metabolic changes in pyrethrum. Moreover, the expression of MYC, the core factor of the JA signal transduction pathway, was significantly up-regulated after MeJA treatment. In the present study, a MYC2-like TF named TcbHLH14 was isolated and cloned from *T. cinerariifolium*. MYC2 was reported to regulate the synthesis of plant secondary metabolites by activating the expression of its target genes. In *Aquilaria sinensis*, AsMYC2 regulates the biosynthesis of sesquiterpene by controlling the expression of the *ASS1* gene [25]. In response to JA, MdMYC2 could significantly up-regulate the transcription level of the anthocyanin biosynthesis gene and increase the content of anthocyanin in apples (*Malus domestica*) [26]. In view of the important role of the TF MYC2 in the synthesis of secondary metabolites induced by JA, it is speculated that pyrethrum *TcbHLH14* may also induce the transcription of key genes in the pyrethrins metabolic pathway to consequently regulate pyrethrins synthesis.

We found that TcbHLH14 exerts this regulatory role by binding to the E-box or G-box region in the target gene promoter. TFs increase or weaken the target gene transcription mainly by specifically recognizing the binding motif in the promoter. E-box (CANNTG) or G-box (CACGTG) is the key element of transcriptional regulation of bHLH proteins [15]. The bHLH_MYC transcription factor TwMYC2a/b of *Tripterygium wilfordii* regulates triptolide synthesis by binding to E-box (CACATG) and T/G-box (CACGTT) motifs in the *TwTPS27a/b* promoter [27]. *S. miltiorrhiza* SmbHLH3 can specifically recognize and bind the G-box of the promoters of *TAT*, *HPPR*, *KSL1*, and *CYP76AH1*, which are key genes in the synthesis of phenolic acid and tanshinone [28].

The promoter sequences of genes in the pyrethrins biosynthesis pathway were isolated and cloned in our laboratory. Further analysis revealed that the *TcAOC* and *TcGLIP* promoters contained six and eight bHLH TF-binding elements, respectively. Through EMSA and Y1H assays, we further found that TcbHLH14 could bind directly to the G-box and E-box motif in the *TcAOC* and *TcGLIP* promoter, respectively. The results of the luciferase reporter assay further showed that TcbHLH14 could activate the expression of the *TcAOC* and *TcGLIP* promoters. These results suggest that *TcAOC* and *TcGLIP* are the target genes of the TF TcbHLH14. Transient overexpression of TcbHLH14 could effectively up-regulate the expression of the *TcAOC* and *TcGLIP* genes; however, the content of pyrethrins was not significantly up-regulated. The reason may be that TcbHLH14 has a fine regulation function similar to that of AtMYC2 [29], which exerts negative regulation of its own expression. Unlike our previous research on TcMYC2, the results of Y1H and Dual-LUC assays indicate that TcbHLH14 does not exhibit binding activity to the *TcCHS* promoter. Furthermore, while transient overexpression of TcMYC2 in leaves up-regulated *TcCHS* expression by more than 20-fold [30], transient overexpression of TcbHLH14 results in only a marginal increase in *TcCHS* expression. This outcome could potentially lead to a reduction in DMAPP flux towards chrysanthemol, resulting in a lack of significant increase in the final accumulation of pyrethrins. In addition, some studies have found that members of the MYC TF family can co-express and regulate the synthesis of secondary metabolites, as is the case for rice OsMYC2 and the OsMYC2-like proteins OsMYL1 and OsMYL2 (both OsMYC2-like proteins), which promote the synthesis of sakuranetin [31]. Thus, lack of cooperation between TcbHLH14 and other MYC proteins may also be the reason for the lack of an effective increase in pyrethrins content under overexpression, although this hypothesis requires further exploration. Transient silencing of *TcbHLH14* could significantly reduce the expression of the *TcGLIP* gene and the content of pyrethrins, whereas the expression level of the *TcAOC* gene was slightly enhanced, which may be due to the competitive binding of other proteins to *TcbHLH14* and activation of *TcAOC* gene expression. In addition, the expression of the pyrethrins biosynthesis pathway genes *TcALDH1* and *TcCHS* is also regulated by TcbHLH14. In summary, our results demonstrate that TcbHLH14 is a positive regulator to promote pyrethrins biosynthesis.

The synthesis of plant secondary metabolites is closely related to the expression level of a series of key structural enzyme genes, and TFs can accurately regulate structural enzyme genes. In a previous study, *Dianthus caryophyllus* transgenic plants were established with overexpression of the linalool synthase gene of *Clarkia breweri* by transgenic technology. Gas chromatography-mass spectrometry analysis of the petal extract showed no significant difference in the linalool content between overexpression and wild-type plants [32]. This suggests that the transfer of a structural gene alone does not necessarily result in the significant accumulation of bioactive products. TFs can often activate the co-expression of multiple key enzyme-encoding genes in a metabolic pathway, which is more efficient than multiple or co-transformation of one or more synthase genes [33]. For example, transferring the TF AaORA into *A. annua* up-regulated the expression of the structural enzyme genes *ADS*, *CYP71AV1*, and *DBR2*, and significantly increased the contents of artemisinin and dihydroartemisinic acid [34]. *S. miltiorrhiza* SmMYB52 directly binds to and activates the promoters of genes encoding four enzymes in the salvianolic acid B biosynthesis pathway (SmTAT1, Sm4CL9, SmC4H1, SmHPPR1) to promote salvianolic acid B accumulation [35]. In citrus, CsMADS5 positively regulates carotenoid synthesis by promoting the transcription of the *PSY*, *PDS*, and *LCYb1* genes [36]. Similarly, we found that TcbHLH14 can specifically bind to the pyrethrins synthesis pathway genes *TcGLIP* and *TcAOC* to consequently up-regulate their expression. Therefore, using TFs to regulate the biosynthesis of secondary plant metabolites has great potential for producing valuable bioactive compounds.

## 4. Materials and Methods

### 4.1. Sequence and Phylogenetic Analysis

The bHLH amino acid sequence of *Arabidopsis thaliana* was downloaded from the National Center for Biotechnology Information (NCBI) database, and the phylogenetic tree of *TcbHLH14* pyrethrum and Arabidopsis bHLH TFs was constructed by MEGA X v10.1.8 [37], and visualized by the online software iTOL v5 [38].

### 4.2. Plant Materials

The tissue culture seedlings of *T. cinerariifolium* ‘W99′ used in this study are preserved in the plant culture room. The seedlings were cultured under a temperature of 25 ± 2 °C with light for 16 h and dark for 8 h. Late the seedlings were planted in the field of flower base of Huazhong Agricultural University. The flowers at the S1–S6 stages [39] and the leaves at the L1–L3 stages [40] were harvested and stored at −80 °C until analysis. The genotype of ‘W99′ seedlings grown in culture flask for 1 month were sprayed with 5 mL of 2 mM MeJA (Macklin Biochemical Co., Ltd., Shanghai, China) dissolved in 0.8% ethanol, and the leaves were collected in triplicate at 0 (control), 2, 4, 6, 8, 12, and 24 h.

### 4.3. RNA Extraction and Reverse Transcription-Quantitative Polymerase Chain Reaction (RT-qPCR)

Total RNA was extracted from *T. cinerariifolium* using TRIzol Reagent (Invitrogen, CA, USA), and reverse-transcribed to synthesize cDNA using TransScript^®^ One-Step gDNA Removal kit and cDNA Synthesis SuperMix kit (TransGen Biotech, Beijing, China). The *TcbHLH14* gene sequence was screened from the MeJA-treated transcriptome determined in our laboratory, and the specific primers for amplifying the open reading frame (ORF) fragment were designed by Primer Premier 5 v1.0 software.

The cDNA was used as the template for qPCR on the Roche LightCycler^®^ 96 Real-time PCR System (Roche, Basel, Switzerland), using SYBR Premix Ex Taq II kits (Takara, Kusatsu, Japan) with three biological and two technical replicates. The qPCR procedure was similar to that reported previously [41]. *TcGADPH* was used as the internal reference, and the sequences of the specific primers are shown in Supplementary Table S3 The 2^−ΔΔCT^ method was used to analyze the relative gene expression [42].

### 4.4. Subcellular Localization Analysis

After digesting the pSuper1300-green fluorescent protein (GFP) vector with *Swa*1 and *Kpn*1 restriction enzymes, the PCR fragment containing the *TcbHLH14* coding region (without the stop codon) was ligated to the linearized pSuper1300-GFP vector by ClonExpress^®^II One Step Cloning Kit (Vazyme Biotech, Nanjing, China). The recombinant plasmid was introduced into *Agrobacterium tumefaciens* strain GV3101 and then co-transformed into tobacco (*Nicotiana benthamiana*) leaves with a red fluorescent protein (RFP)-tagged nuclear marker. After 72 h of weak light exposure, fluorescence signals were observed with a Leica TCS-SP8 fluorescence microscope (Leica, Wetzlar, Germany).

### 4.5. Yeast One-Hybrid (Y1H) Assay

The *TcbHLH14* sequence was integrated in the pGADT7 vector, which was digested with *Nde*I and *Xho*I restriction enzymes to obtain the pGADT7:*bHLH14* recombinant plasmid. The pH2.1:*TcGLIP* and pH2.1:*TcAOC* vectors were obtained as described previously [30]. The effector and reporter plasmids were co-transformed into the yeast strain Y187 using the Super Yeast Transformation kit (Coolaber, Beijing, China). The transformed yeast cells were coated on a deficient medium supplemented with 3-amino-1,2,4-triazol (3-AT) at various concentrations to determine the appropriate concentration of 3-AT, the transformed yeast cells were selected on DDO (SD/-Leu/-Trp) medium, and the Y1H interaction assay was carried out on 30 °C TDO (SD/-Leu/-Trp/-His) medium for 3 days.

### 4.6. Dual-Luciferase (LUC) Transient Expression Assays

To obtain the recombinant vector, the ORF sequence of *TcbHLH14* was integrated in the *Hind*III linearized pGreenII62-SK vector by the ClonExpress II One Step Cloning Kit. Together with the previously constructed pGreenII0800-LUC:AOC and GLIP vectors [30], the reporter vector was transformed into *A. tumefaciens* GV3101 (containing the pSoup19 vector) and then transformed into *N. benthamiana* leaves. The negative control vector was transformed into the corresponding part of the same leaf. Leaf materials were collected after 48 h under low light. The luminescence was detected under the NightSHADE LB 985 imaging system (Berthold, Bad Wildbad, Germany) by the Dual-Luciferase reporter assay system kit (Promega, Madison, WI, USA).

### 4.7. Electrophoretic Mobility Shift Assay (EMSA)

The ORF of *TcbHLH14* without the stop codon was integrated in *B*amHI and *X*hoI -linearized pET28a vector, which was transformed in the *Escherichia coli* Rosetta (DE3) strain. The recombinant protein was induced by 0.5 mM isopropyl β-d-1-thiogalactopyranoside, incubated at 18 °C for 16 h, and purified on a Ni-NTA column. The *cis*-elements (E-box motif, G-box motif) in the *TcAOC* and *TcGLIP* promoter fragments were labeled at the 5′ end with the fluorophore 6-FAM, and 50-fold of unlabeled wild-type and *cis*-element mutant DNA fragments were used as competitors. EMSA detection was then performed as described previously [43].

### 4.8. Virus-Induced Gene Silencing (VIGS)

After linearizing the pTRV2 vector with *Bam*HI enzyme, The 276-bp specific fragment from *TcbHLH14* was designed by siRNA Finder software [44], and integrated into the pTRV2 vector by homologous recombination, and the obtained plasmids pTRV2:*TcbHLH14* and pTRV1 were co-transformed into the chemically active *A. tumefaciens* GV3101 strain. The VIGS experiment was carried out in *T. cinerariifolium* as reported previously [45]. Co-transformation of the empty vectors pTRV1 and pTRV2 was used as a control. After 14 days, the samples were used for RT-qPCR and high-performance liquid chromatography (HPLC) analyses.

### 4.9. Transient Overexpression of TcbHLH14

The expression vector pGreenII62-SK was driven by the CaMV35S promoter and the constructed pGreenII62-SK:*TcbHLH14* vector was transformed into *A. tumefaciens* GV3101 (containing the pSoup19 vector). Transient transformation of *T. cinerariifolium* leaves was performed as described previously [46]. After the RT-qPCR assay, 4-day-old leaves were taken for HPLC analysis.

### 4.10. Pyrethrin Content Measurement by HPLC

The pyrethrins were extracted as described previously [47] and filtered with a 0.22-µm mesh filter, then determined using the Waters HPLC system (Waters Corporation, Milford, MA, USA) equipped with a photodiode array detector using three biological replicates as described previously [48]. pyrethrum extract (Sigma-Aldrich, St. Louis, MO, USA) was used as the standard.

### 4.11. Statistical Analysis

All experiments were performed with at least three biological replicates. One-way ANOVA and Student’s *t*-test (or Tukey post hoc test) were performed using SPSS v.20 software. *p*-values < 0.05 were considered statistically significant.

## Data Availability

Not applicable.

## Supplementary Materials

The following supporting information can be downloaded at: https://www.mdpi.com/article/10.3390/ijms24087379/s1.

## References

[1] Casida J.E., Quistad G.B. (1995). Pyrethrum Flowers: Production, Chemistry, Toxicology, and Uses.

[2] Jeran N., Grdiša M., Varga F., Šatović Z., Liber Z., Dabić D., Biošić M. (2021). Pyrethrin from Dalmatian pyrethrum (*Tanacetum cinerariifolium*/Trevir./Sch. Bip.): Biosynthesis, biological activity, methods of extraction and determination. Phytochem. Rev..

[3] Grdisa M., Jeran N., Varga F., Klepo T., Nincevic T., Satovic Z. (2022). Accumulation patterns of six pyrethrin compounds across the flower developmental stages-comparative analysis in six natural dalmatian pyrethrum populations. Agronomy.

[4] Lybrand D.B., Xu H., Last R.L., Pichersky E. (2020). How plants synthesize pyrethrins: Safe and biodegradable insecticides. Trends Plant Sci..

[5] Matsuda K., Kikuta Y., Haba A., Nakayama K., Katsuda Y., Hatanaka A., Komai K. (2005). Biosynthesis of pyrethrin I in seedlings of *Chrysanthemum cinerariaefolium*. Phytochemistry.

[6] Hu H., Li J.J., Delatte T., Vervoort J., Gao L., Verstappen F., Xiong W., Gan J., Jongsma M.A., Wang C.Y. (2018). Modification of chrysanthemum odour and taste with chrysanthemol synthase induces strong dual resistance against cotton aphids. Plant Biotechnol. J..

[7] Xu H., Moghe G.D., Wiegert-Rininger K., Schilmiller A.L., Barry C.S., Last R.L., Pichersky E. (2018). Coexpression analysis identifies two oxidoreductases involved in the biosynthesis of the monoterpene acid moiety of natural pyrethrin insecticides in *Tanacetum cinerariifolium*. Plant Physiol..

[8] Xu H., Li W., Schilmiller A.L., van Eekelen H., de Vos R.C.H., Jongsma M.A., Pichersky E. (2019). Pyrethric acid of natural pyrethrin insecticide: Complete pathway elucidation and reconstitution in *Nicotiana benthamiana*. New Phytol..

[9] Matsui R., Takiguchi K., Kuwata N., Oki K., Takahashi K., Matsuda K., Matsuura H. (2020). Jasmonic acid is not a biosynthetic intermediate to produce the pyrethrolone moiety in pyrethrin II. Sci. Rep..

[10] Wasternack C., Strnad M. (2019). Jasmonates are signals in the biosynthesis of secondary metabolites- Pathways, transcription factors and applied aspects-A brief review. New Biotechnol..

[11] Yoeun S., Cho K., Han O. (2018). Structural evidence for the substrate channeling of rice allene oxide cyclase in biologically analogous Nazarov reaction. Front. Chem..

[12] Li W., Zhou F., Pichersky E. (2018). Jasmone hydroxylase, a key enzyme in the synthesis of the alcohol moiety of pyrethrin insecticides. Plant Physiol..

[13] Li W., Lybrand D.B., Zhou F., Last R.L., Pichersky E. (2019). Pyrethrin biosynthesis: The cytochrome P450 oxidoreductase CYP82Q3 converts jasmolone to pyrethrolone. Plant Physiol..

[14] Kikuta Y., Ueda H., Takahashi M., Mitsumori T., Yamada G., Sakamori K., Takeda K., Furutani S., Nakayama K., Katsuda Y. (2012). Identification and characterization of a GDSL lipase-like protein that catalyzes the ester-forming reaction for pyrethrin biosynthesis in *Tanacetum cinerariifolium*- a new target for plant protection. Plant J..

[15] Carretero-Paulet L., Galstyan A., Roig-Villanova I., Martinez-Garcia J.F., Bilbao-Castro J.R., Robertson D.L. (2010). Genome-wide classification and evolutionary analysis of the bHLH family of transcription factors in Arabidopsis, poplar, rice, moss, and algae. Plant Physiol..

[16] Heim M.A., Jakoby M., Werber M., Martin C., Weisshaar B., Bailey P.C. (2003). The basic helix-loop-helix transcription factor family in plants: A genome-wide study of protein structure and functional diversity. Mol. Biol. Evol..

[17] Fernández-Calvo P., Chini A., Fernández-Barbero G., Chico J.-M., Gimenez-Ibanez S., Geerinck J., Eeckhout D., Schweizer F., Godoy M., Franco-Zorrilla J.M. (2011). The Arabidopsis bHLH transcription factors MYC3 and MYC4 are targets of JAZ repressors and act additively with MYC2 in the activation of jasmonate responses. Plant Cell.

[18] Pérez-Alonso M.-M., Sánchez-Parra B., Ortiz-García P., Santamaría M.E., Díaz I., Pollmann S. (2021). Jasmonic acid-dependent myc transcription factors bind to a tandem g-box motif in the yucca8 and yucca9 promoters to regulate biotic stress responses. Int. J. Mol. Sci..

[19] Goklany S., Rizvi N.F., Loring R.H., Cram E.J., Lee-Parsons C.W.T. (2013). Jasmonate-Dependent Alkaloid Biosynthesis in Catharanthus roseus Hairy Root Cultures Is Correlated with the Relative Expression of Orca and Zct Transcription Factors. Biotechnol. Prog..

[20] Alfieri M., Vaccaro M.C., Cappetta E., Ambrosone A., De Tommasi N., Leone A. (2018). Coactivation of MEP-biosynthetic genes and accumulation of abietane diterpenes in *Salvia sclarea* by heterologous expression of WRKY and MYC2 transcription factors. Sci. Rep..

[21] Xiang L., Jian D., Zhang F., Yang C., Bai G., Lan X., Chen M., Tang K., Liao Z. (2019). The cold-induced bHLH transcription factor AabHLH112 promotes artemisinin biosynthesis in Artemisia annua. J. Exp. Bot..

[22] Ji Y.P., Xiao J.W., Shen Y.L., Ma D.M., Li Z.Q., Pu G.B., Li X., Huang L.L., Liu B.Y., Ye H.C. (2014). Cloning and Characterization of AabHLH1, a bHLH Transcription Factor that Positively Regulates Artemisinin Biosynthesis in Artemisia annua. Plant Cell Physiol..

[23] Yang N., Zhou W., Su J., Wang X., Li L., Wang L., Cao X., Wang Z. (2017). Overexpression of SmMYC2 increases the production of phenolic acids in Salvia miltiorrhiza. Front. Plant Sci..

[24] Dabiri M., Majdi M., Bahramnejad B. (2020). Partial sequence isolation of DXS and AOS genes and gene expression analysis of terpenoids and pyrethrin biosynthetic pathway of *Chrysanthemum cinerariaefolium* under abiotic elicitation. Acta Physiol. Plant..

[25] Xu Y.H., Liao Y.C., Lv F.F., Zhang Z., Sun P.W., Gao Z.H., Hu K.P., Sui C., Jin Y., Wei J.H. (2017). Transcription factor AsMYC2 controls the jasmonate-responsive expression of ASS1 regulating sesquiterpene biosynthesis in *Aquilaria sinensis* (Lour.) Gilg. Plant Cell Physiol..

[26] An J.P., Li H.H., Song L.Q., Su L., Liu X., You C.X., Wang X.F., Hao Y.J. (2016). The molecular cloning and functional characterization of MdMYC2, a bHLH transcription factor in apple. Plant Physiol. Biochem..

[27] Huo Y.B., Zhang J., Zhang B., Chen L., Zhang X., Zhu C.A.S. (2021). MYC2 Transcription Factors TwMYC2a and TwMYC2b Negatively Regulate Triptolide Biosynthesis in *Tripterygium wilfordii* Hairy Roots. Plants.

[28] Zhang C., Xing B., Yang D., Ren M., Guo H., Yang S., Liang Z. (2020). SmbHLH3 acts as a transcription repressor for both phenolic acids and tanshinone biosynthesis in Salvia miltiorrhiza hairy roots. Phytochemistry.

[29] Dombrecht B., Xue G.P., Sprague S.J., Kirkegaard J.A., Ross J.J., Reid J.B., Fitt G.P., Sewelam N., Schenk P.M., Manners J.M. (2007). MYC2 differentially modulates diverse jasmonate-dependent functions in *Arabidopsis*. Plant Cell.

[30] Zeng T., Li J.-W., Xu Z.-Z., Zhou L., Li J.-J., Yu Q., Luo J., Chan Z.-L., Jongsma M.A., Hu H. (2022). TcMYC2 regulates Pyrethrin biosynthesis in *Tanacetum cinerariifolium*. Hortic. Res..

[31] Ogawa S., Miyamoto K., Nemoto K., Sawasaki T., Yamane H., Nojiri H., Okada K. (2017). OsMYC2, an essential factor for JA-inductive sakuranetin production in rice, interacts with MYC2-like proteins that enhance its transactivation ability. Sci. Rep..

[32] Lavy M., Zuker A., Lewinsohn E., Larkov O., Ravid U., Vainstein A., Weiss D. (2002). Linalool and linalool oxide production in transgenic carnation flowers expressing the Clarkia breweri linalool synthase gene. Mol. Breed..

[33] Sun M., Shi M., Wang Y., Huang Q., Yuan T., Wang Q., Wang C., Zhou W., Kai G. (2019). The biosynthesis of phenolic acids is positively regulated by the JA-responsive transcription factor ERF115 in Salvia miltiorrhiza. J. Exp. Bot..

[34] Lu X., Zhang L., Zhang F., Jiang W., Shen Q., Zhang L., Lv Z., Wang G., Tang K. (2013). A a ORA, a trichome-specific AP2/ERF transcription factor of *Artemisia annua*, is a positive regulator in the artemisinin biosynthetic pathway and in disease resistance to *Botrytis cinerea*. New Phytol..

[35] Yang R., Wang S., Zou H., Li L., Li Y., Wang D., Xu H., Cao X. (2021). R2R3-MYB transcription factor SmMYB52 positively regulates biosynthesis of salvianolic acid B and inhibits root growth in Salvia miltiorrhiza. Int. J. Mol. Sci..

[36] Lu S., Ye J., Zhu K., Zhang Y., Zhang M., Xu Q., Deng X. (2021). A fruit ripening-associated transcription factor CsMADS5 positively regulates carotenoid biosynthesis in citrus. J. Exp. Bot..

[37] Kumar S., Stecher G., Li M., Knyaz C., Tamura K. (2018). MEGA X: Molecular evolutionary genetics analysis across computing platforms. Mol. Biol. Evol..

[38] Letunic I., Bork P. (2021). Interactive Tree Of Life (iTOL) v5: An online tool for phylogenetic tree display and annotation. Nucleic Acids Res..

[39] Ramirez A.M. (2013). Pyrethrum Secondary Metabolism: Biosynthesis, Localization and Ecology of Defence Compounds. Ph.D. Thesis.

[40] Zito S.W., Zieg R.G., Staba E.J. (1983). Distribution of pyrethrins in oil glands and leaf tissue of *Chrysanthemum cinerariaefolium*. Planta Med..

[41] Zeng T., Li J.-W., Zhou L., Xu Z.-Z., Li J.-J., Hu H., Luo J., Zheng R.-R., Wang Y.-Y., Wang C.-Y. (2021). Transcriptional responses and GCMS analysis for the biosynthesis of pyrethrins and volatile terpenes in *Tanacetum coccineum*. Int. J. Mol. Sci..

[42] Livak K.J., Schmittgen T.D. (2001). Analysis of relative gene expression data using real-time quantitative PCR and the 2^−ΔΔCT^ method. Methods.

[43] Xu H., Luo D., Zhang F. (2021). DcWRKY75 promotes ethylene induced petal senescence in carnation (*Dianthus caryophyllus* L.). Plant J..

[44] Lück S., Kreszies T., Strickert M., Schweizer P., Kuhlmann M., Douchkov D. (2019). siRNA-Finder (si-Fi) Software for RNAi-Target Design and Off-Target Prediction. Front. Plant Sci..

[45] Senthil-Kumar M., Mysore K.S. (2014). Tobacco rattle virus-based virus-induced gene silencing in *Nicotiana benthamiana*. Nat. Protoc..

[46] Zhou L., Li J., Zeng T., Xu Z., Luo J., Zheng R., Wang Y., Wang C. (2022). TcMYB8, a R3-MYB transcription factor, positively regulates pyrethrin biosynthesis in *Tanacetum Cinerariifolium*. Int. J. Mol. Sci..

[47] Li J., Jongsma M.A., Wang C.Y. (2014). Comparative analysis of pyrethrin content improvement by mass selection, family selection and polycross in pyrethrum [*Tanacetum cinerariifolium* (Trevir.) Sch.Bip.] populations. Ind. Crops Prod..

[48] Xu Z., Zeng T., Li J., Zhou L., Li J., Luo J., Zheng R., Wang Y., Hu H., Wang C. (2023). TcbZIP60 positively regulates pyrethrins biosynthesis in *Tanacetum cinerariifolium*. Front. Plant Sci..

