# Supplementary material for: TcbHLH14 a Jasmonate Associated MYC2-like Transcription Factor Positively Regulates Pyrethrin Biosynthesis in Tanacetum cinerariifolium
Source: Int J Mol Sci. 2023 Apr 17;24(8):7379. doi: 10.3390/ijms24087379 (PMC10138541; doi:10.3390/ijms24087379)
Supplement: Supplementary file 1 [file ijms-24-07379-s001.zip › ijms-2341090-supplementary.pdf]

Supplemental information

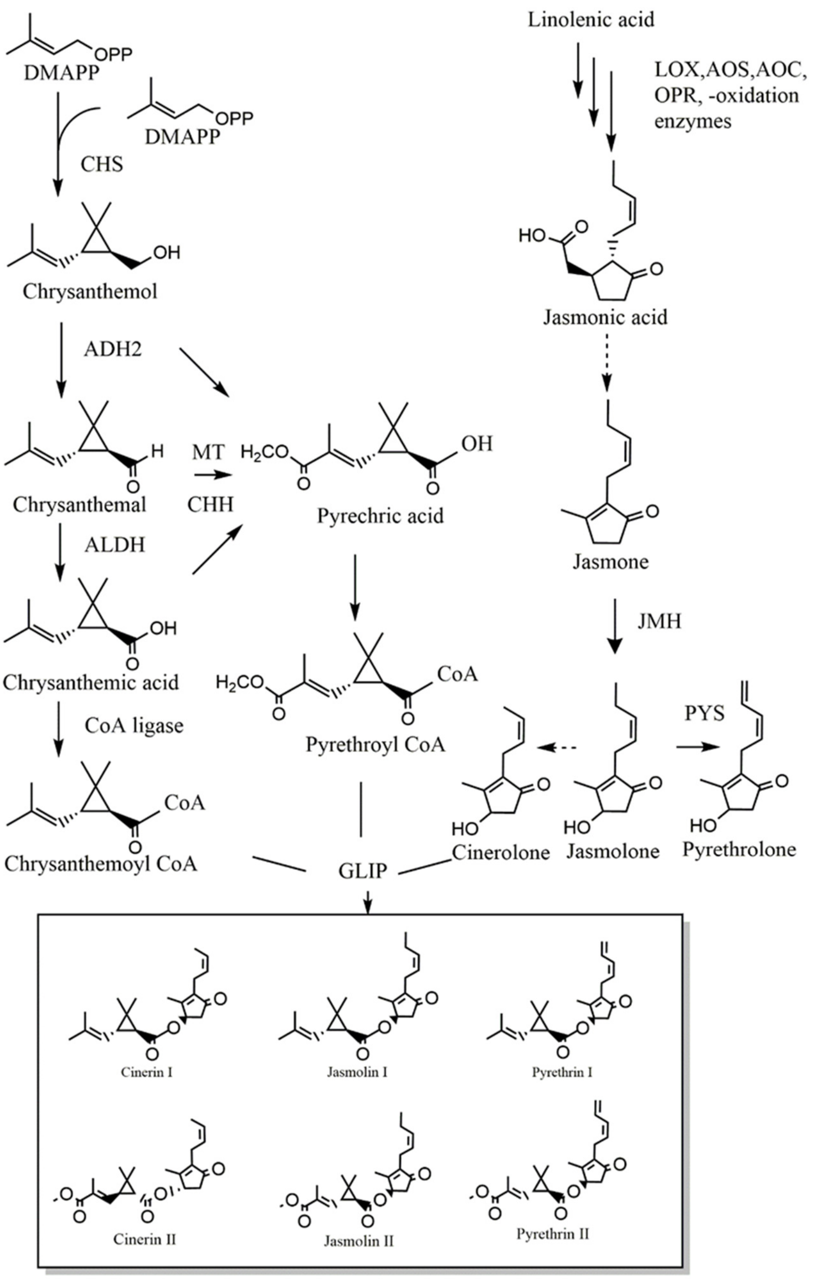

Figure S1. The biosynthesis pathway of pyrethrins

The solid box encloses the six major pyrethrins. Abbreviations: ADH2, alcohol dehydrogenase2; ALDH, aldehyde dehydrogenase; CHH, chrysanthemol 10-hydroxylase; CHS, chrysanthemol synthase; GLIP, GDSL lipase-like protein; JMH, jasmolone hydroxylase; MT, 10-carboxychrysanthemic acid 10-methyltransferase; PYS, pyrethrolone synthase.

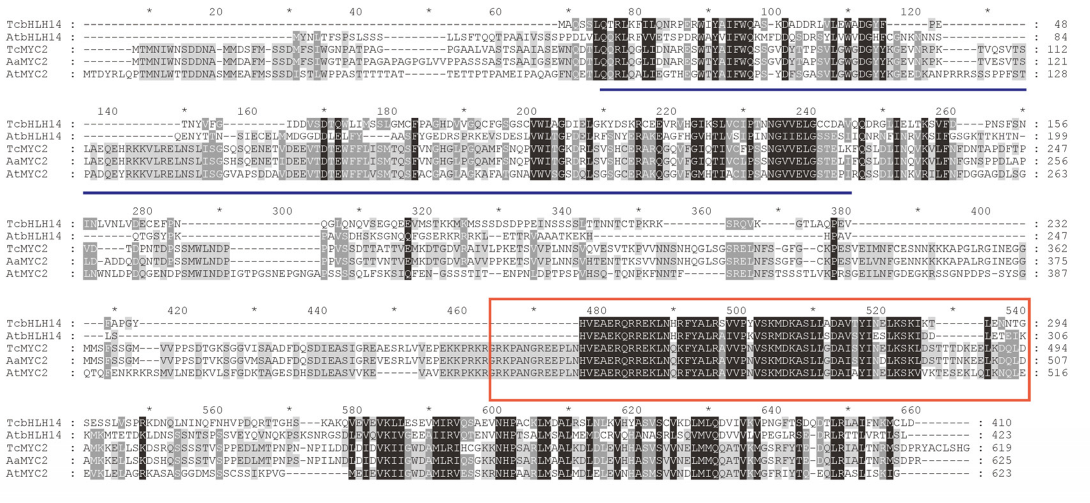

Figure S2. Multiple amino acid sequence alignment of TcbHLH14 and bHLH_MYC proteins of other plants.
Consistent sequences are marked in white text with a black background. The HLH domain is boxed in pink and the bHLH-MYC_N domain is lined in blue. TcbHLH14 is marked with a red triangle.

Table S1. Content of pyrethrins in leaves after transient overexpression of TcbHLH14 for 4 days.

Pyrethrins	OE				CK			
	Retention time		Peak area		Retention time		Peak area	
	Time	SD	Area	SD	Time	SD	Area	SD
Cinerin II	7.973	0.004	432303.667	20029.579	7.998	0.003	380037.667	8737.604
Pyrethrin II	8.675	0.005	1508409.333	62410.078	8.695	0.005	1707882.667	39671.574
Jasmolin II	10.029	0.006	143267.333	6197.035	10.101	0.004	150520.667	3355.006
Cinerin I	14.725	0.049	336471.667	23964.212	14.785	0.019	188701.000	11726.579
Pyrethrin I	16.184	0.022	2722187.333	107978.294	16.244	0.015	2480796.000	55588.480
Jasmolin I	19.039	0.026	43336.667	4516.825	19.069	0.016	21074.667	610.786
Total pyrethrins	/	/	5185976.000	219383.699	/	/	4929012.667	107652.998

Table S2. Content of pyrethrins in leaves after transient silencing of TcbHLH14.

Pyrethrins	VIGS				CK			
	Retention time		Peak area		Retention time		Peak area	
	Time	SD	Area	SD	Time	SD	Area	SD
Cinerin Ⅱ	8.010	0.001	113512.333	2809.189	8.010	0.003	230706.333	10006.899
Pyrethrin Ⅱ	8.692	0.007	900216.667	24653.002	8.683	0.001	1714069.333	46990.546
Jasmolin Ⅱ	10.056	0.007	55185.667	4682.275	10.020	0.005	100661.000	5917.866
Cinerin Ⅰ	14.790	0.021	57306.667	6864.298	14.806	0.027	60465.667	6122.550
Pyrethrin Ⅰ	16.271	0.013	1099836.333	105169.763	16.268	0.012	1585931.667	75813.527
Jasmolin Ⅰ	19.178	0.005	22940.000	3082.619	19.188	0.026	24927.000	2799.476
Total pyrethrins	/	/	2248997.667	130755.696	/	/	3716761.000	134269.885

Table S3. Primers used in this study.

ID	Primer name	Primer sequences(5' to 3')
Cloning	TcbHLH14_F	ATGGCACAATCCTCTCTTCAAAC
Cloning	TcbHLH14_R	TTAATCCAAGCACATTTTGTTAA
Realtime PCR	TcGAPDH_RT_F	AAGGAGGAATCTGAAGGAAAGCTG
Realtime PCR	TcGAPDH_RT_R	GTTGTTGTTCAAAGCGATTCCAGC
Realtime PCR	TcbHLH14_RT_F	GCACTTAGGAGCGTAGTTCCATAC
Realtime PCR	TcbHLH14_RT_R	TTGATTGATGTTCAGTTGGTTGTCC
Realtime PCR	TcCHS_RT_F	ACGTGCATCTTCTGGACCTCTTC
Realtime PCR	TcCHS_RT_R	TGAACAATCCGACGGTTAAGAGTC
Realtime PCR	TcGLIP_RT_F	GCCGGGAATGCGAGCAAAACAAC
Realtime PCR	TcGLIP_RT_R	CGCTCTCGCCTTCCTTAAAACCATA
Realtime PCR	TcALDH_RT_F	CATTCCGCTACTTTGCTGGTGC
Realtime PCR	TcALDH_RT_R	TCCAAGGAATGATGTGTCCAACTAC
Realtime PCR	TcAOC_RT_F	TTCAAGGCGCATACATCACAACT
Realtime PCR	TcAOC_RT_R	GTCACCGGGGTAACTAACAACTCA
linking to pGADT7	TcbHLH14_pGADT7_F	gtaccagattacgcTcatatgATGGCACAATCCTCTCTTCAAAC
linking to pGADT7	TcbHLH14_pGADT7_R	acgatTcaTctgcagcTcgagTTAATCCAAGCACATTTTGTTAAAAAT
linking to pHis2.1	TcAOC_pHis2.1_F	gactcactatagggcgaattcTATTTAACTTGTATATATACATGGGTTGAAGC
linking to pHis2.1	TcAOC_pHis2.1_R	attactagtggatccacgcgTAGTTGTTAAGATTTGTTTTAATGTTTAATGC
linking to pHis2.1	TcGLIP_pHis2.1_F	gactcactatagggcgaattcAAACTAGAAGCAAAGATCATCGTACTTC
linking to pHis2.1	TcGLIP_pHis2.1_R	attactagtggatccacgcgTAGCTTATATGTGCTCAGACAAGAGGT
linking to pSuper1300GFP	TcbHLH14-pS1300-F	gggcccggggTcgacatttaaatATGGCACAATCCTCTCTTCAAAC
linking to pSuper1300GFP	TcbHLH14-pS1300-R	gcccttgcTcaccatggtaccATCCAAGCACATTTTGTTAAAAATCG
linking to pGreenIISK62	TcbHLH14_SK62_F	caggaattcgatatcaagcttATGGCACAATCCTCTCTTCAAAC
linking to pGreenIISK62	TcbHLH14_SK62_R	gtcgacggtatcgataagcttTTAATCCAAGCACATTTTGTTAA
linking to pGreenII0800-LUC	TcAOC_pGreenII0800-LUC_F	gtcgacggtatcgataagcttTATTTAACTTGTATATATACATGGGTTGAAGC
linking to pGreenII0800-LUC	TcAOC_pGreenII0800-LUC_R	caggaattcgatatcaagctTAGTTGTTAAGATTTGTTTTAATGTTTAATGC
linking to pGreenII0800-LUC	TcGLIP_pGreenII0800-LUC_F	gtcgacggtatcgataagcttAAACTAGAAGCAAAGATCATCGTACTTC
linking to pGreenII0800-LUC	TcGLIP_pGreenII0800-LUC_R	caggaattcgatatcaagctTAGCTTATATGTGCTCAGACAAGAGGT
linking to pET28a	TcbHLH14_pET28a_F	cagcaaatgggTcgcggaTccATGGCACAATCCTCTCTTCAAACCCG
linking to pET28a	TcbHLH14_pET28a_R	gtggtggtggtggtgcTcgagATCCAAGCACATTTTGTTAAAAATCGCAAG
Probes used in EMSA	Probe_GLIP_F	CCTGACAGTTGCTATTTAGTGCTGTCTACTTGTTTAGTTGTGGAGCAAATGACT
Probes used in EMSA	Probe_GLIP_R	AGTCATTTGCTCCACAACTAAACAAGTAGACAGCACTAAATAGCAACTGTCAGG
Probes used in EMSA	Probe_AOC_F	CAACTAATCACACACCGCCACGTGTCCACCGTCCACCTACT
Probes used in EMSA	Probe_AOC_R	AGTAGGTGGACGGTGGACACGTGGCGGTGTGTGATTAGTTG
Probes used in EMSA	mProbe_AOC_F	CAACTAATCACACACCGCTCTTCTTCCACCGTCCACCTACT
Probes used in EMSA	mProbe_AOC_R	AGTAGGTGGACGGTGGAAGAAGAGCGGTGTGTGATTAGTTG
Probes used in EMSA	mProbe_GLIP_F	CCTGATCTCGTCTATTTAGTGCTGTCTACTTGTTTAGTTGTGGAGTCCCGTACT
Probes used in EMSA	mProbe_GLIP_R	AGTACGGGACTCCACAACTAAACAAGTAGACAGCACTAAATAGACGAGATCAGG
linking to pTRV2	TcbHLH14_VIGS_F	agaaggccTccatggggaTccGGACAACCAACTGAACATCAATCA
linking to pTRV2	TcbHLH14_VIGS_R	cgtgagcTcggtaccggaTccTCGCAAGTCGTAAGGTGTCTTG
